# Higher Plant Cytochrome *b*5 Polypeptides Modulate Fatty Acid Desaturation

**DOI:** 10.1371/journal.pone.0031370

**Published:** 2012-02-23

**Authors:** Rajesh Kumar, Lam-Son Phan Tran, Anjanasree K. Neelakandan, Henry T. Nguyen

**Affiliations:** Division of Plant Sciences and National Center for Soybean Biotechnology, University of Missouri, Columbia, Missouri, United States of America; Max Planck Institute for Chemical Ecology, Germany

## Abstract

**Background:**

Synthesis of polyunsaturated fatty acids (PUFAs) in the endoplasmic reticulum of plants typically involves the fatty acid desaturases FAD2 and FAD3, which use cytochrome b_5_ (Cb5) as an electron donor. Higher plants are reported to have multiple isoforms of Cb5, in contrast to a single Cb5 in mammals and yeast. Despite the wealth of information available on the roles of FAD2 and FAD3 in PUFA synthesis, information regarding the contributions of various Cb5 isoforms in desaturase-mediated reactions is limited.

**Results:**

The present functional characterization of Cb5 polypeptides revealed that all *Arabidopsis* Cb5 isoforms are not similarly efficient in ω-6 desaturation, as evidenced by significant variation in their product outcomes in yeast-based functional assays. On the other hand, characterization of Cb5 polypeptides of soybean (*Glycine max*) suggested that similar ω-6 desaturation efficiencies were shared by various isoforms. With regard to ω-3 desaturation, certain *Cb5* genes of both *Arabidopsis* and soybean were shown to facilitate the accumulation of more desaturation products than others when co-expressed with their native *FAD3*. Additionally, similar trends of differential desaturation product accumulation were also observed with most *Cb5* genes of both soybean and *Arabidopsis* even if co-expressed with non-native *FAD3*.

**Conclusions:**

The present study reports the first description of the differential nature of the *Cb5* genes of higher plants in fatty acid desaturation and further suggests that ω-3/ω-6 desaturation product outcome is determined by the nature of both the Cb5 isoform and the fatty acid desaturases.

## Introduction

In higher eukaryotes, cytochrome b5 (Cb5) is a small heme-binding protein typically associated with endoplasmic reticulum (ER) and outer mitochondrial membranes. In higher plants, animals and fungi, the ER-resident Cb5 provides electrons for the desaturation of acyl-CoA fatty acids (FAs) [1]. In higher plants, Cb5 has also been implicated as an electron donor in fatty acid hydroxylation [2], [3], [4], triple bond formation [5], [6], sphingolipid long-chain base hydroxylation and desaturation [7], [8], sterol desaturation [9] and cytochrome P450-mediated reactions [10]. Apart from roles in lipid metabolism, it has recently been reported that interaction with the ER-resident Cb5 increases the affinity of plasma membrane-associated sucrose and sorbitol transporters for their substrates, which is critical for adjusting sugar level in cells [11].

In higher plants, *de novo* synthesis of FAs occurs in plastids, but their subsequent desaturation occurs in either plastids or ER. The introduction of double bonds in fatty acids absolutely requires oxygen and co-factors that provide electrons to the desaturases housed in these two compartments. In plastids, reduced ferredoxin provides electrons to desaturases, whereas in ER Cb5 donates electrons to both FAD2 and FAD3 [12]. Although the relative contribution of the two pathways to total cellular desaturation products varies with tissues and species, synthesis of most PUFAs occurs by desaturases residing in the ER, namely FAD2 (18∶1 to 18∶2 desaturation) and FAD3 (18∶2 to 18∶3 desaturation) [13], [14].

Higher plants including tobacco [15], [16], *Arabidopsis* [4], [17], tung [18], *Crepis alpina* [6], and soybean (this study) are uniquely endowed with multiple *Cb5* genes, as opposed to the single *Cb5* gene found in mammals [19] and yeast [20]. In addition to the typical ‘independent’ Cb5, a Cb5-like domain has been reported in many front-end desaturases, such as the Δ^5^ or Δ^6^ desaturases of mammals and the nematode *Caenorhabditis elegans* [7] and the Δ^6^-desaturase of borage plants [21]. In higher plants, the Cb5 motif has been reported in the sphingolipid Δ^8^ –LCB desaturase [7] and nitrate reductase [22]. Angiosperms encode multiple independent Cb5 isoforms and FAD2 and FAD3 genes that lack Cb5-like domains. While FAD2 and FAD3 have been extensively characterized, the relationships between the various Cb5 isoforms and the desaturases are not defined, particularly with regard to PUFA synthesis.

To understand the contributions of the Cb5 isoforms in FAD2 and FAD3 enzymatic reactions, we characterized different *Cb5* genes from both soybean and *Arabidopsis* using a mutant yeast strain deficient in its endogenous *Cb5* gene. Yeast has proven to be an excellent experimental system for functional characterization of plant lipid metabolism-associated genes, such as desaturases [23], [24], [25], [26], conjugases [27], epoxygenases [28], hydroxylases [3], [29], Cb5 [9] and NADH: cytochrome b5 reductase [4], [30]. Here, we report that expression of various soybean Cb5 isoforms led to similar accumulation of the linoleic acid (18∶2), product of FAD2, and that expression of the various *Arabidopsis* Cb5 isoforms displayed significant variation in 18∶2 levels. The apparent non-discriminatory ω-6 desaturation product outcome demonstrated by soybean Cb5 isoforms is discussed. Co-expression of certain *Cb5* genes from both soybean and *Arabidopsis* with their native *FAD3*, resulted in the significant accumulation of the α-linolenic acid (18∶3) product. Moreover, the differential ω-3 desaturation properties associated with such *Cb5*s were also observed when co-expressed with non-native *FAD3*.

## Results

### Identification, cloning and expression pattern of soybean *Cb5* genes

**Figure 1:**
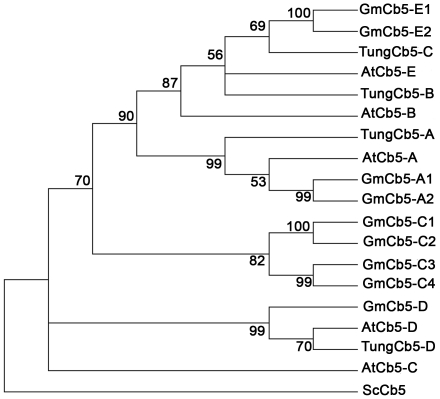
Phylogenetic relationship of soybean Cb5 proteins. Accession numbers of soybean Cb5-encoding genes are provided in Table 1. Accession numbers of *Arabidopsis* Cb5-encoding genes: *AtCb5-A* (*At5g53560*), *AtCb5-B* (*At5g48810*), *AtCb5-C* (*At2g46650*) and *AtCb5-E* (*At2g32720*). Accession numbers of tung Cb5-encoding genes: *TungCb5-A* (AY578727), *TungCb5-B* (AY578728) and *TungCb5-C* (AY578729). The percentage of bootstrap values (1000 replicates) is shown at the branch nodes. Cb5 = Cytochrome b5; At = *Arabidopsis thaliana*; Gm = *Glycine max*; Sc = *Saccharomyces cerevisiae*).

The first public release of a nearly complete genome sequence of soybean (*Glycine max* var. Williams 82; Glyma0 model; www.phytozome.org) enabled the identification of *Cb5* genes on a genome-wide scale. When the deduced protein sequence of *Arabidopsis Cb5* (*At5g53560*) was used to query (TBLASTN) the whole soybean genome, eleven *Cb5* candidates were identified (Table 1). Different soybean *Cb5* (*GmCb5*) genes were named based on phylogenetic clustering of their predicted proteins with those of *Arabidopsis* and tung Cb5 proteins (Figure 1; the GmCb5-B1 and –B2 has been removed, for explanation see below). The predicted protein sequences of *GmCb5* ORFs in the Glyma0 model all contained the heme-binding motif (–HPGGD-), essential for Cb5 function (data not shown). All GmCb5 proteins clustered with the known ER-targeted Cb5 proteins of tung [18] and *Arabidopsis* [31], except GmCb5-D (Figure 1). The GmCb5-D clustered with the known outer mitochondrial membrane-targeted Cb5 protein of tung [18] and *Arabidopsis* [31].

**Table 1:** Cytochrome *b*5 (*Cb5*) genes of soybean.

Accession number (Glyma0 model)	CDS (bp) (Glyma0 model)	Named as (this study)	Accession number (Glyma1 model)
Gm0096x00349.2	405	GmCb5-A1	Glyma06g13840.1
Gm0065x00536.3	405	GmCb5-A2	Glyma04g41010.4
Gm0119x00133	429	GmCb5-B1	-
Gm0151x00024	417	GmCb5-B2	-
Gm0024x00061	408	GmCb5-E1	Glyma03g13520.1
Gm0037x00062	408	GmCb5-E2	Glyma18g30680.1
Gm0149x00147.2	429	GmCb5-C1	Glyma07g05830.1
Gm0161x00053.2	417	GmCb5-C2	Glyma16g02410.1
Gm0041x00751	426	GmCb5-C3	Glyma19g44780.1
Gm0076x00051.1	426	GmCb5-C4	Glyma03g42070.1
Gm0096x00363	366	GmCb5-D	Glyma06g13710.1

The *GmCb5* genes could be amplified by PCR, except *GmCb5-B1* and *GmCb5-B2*. A search in the publicly available soybean EST datasets [32] identified ESTs of all *GmCb5* genes except *GmCb5-B1* and *GmCb5-B2* (data not shown), which led us to believe that these two could be pseudogenes. In addition, in the later version of the soybean genome model, (Glyma1), the annotation for both *GmCb5-B1* and *GmCb5-B2* were absent, consistent with conclusion that these two genes were perhaps wrongly annotated in previous genome model.

The nucleotide sequences of the cloned cDNAs of *Cb5* genes *GmCb5-A1*, *GmCb5-E1*, *GmCb5-E2*, *GmCb5-C1*, *GmCb5-C2* and *GmCb5-C3* were 100% identical to the published sequences (www.phytozome.org, Glyma1 model). The two gene pairs *GmCb5-E1* and *GmCb52-E2* and *GmCb5-C1* and*GmCb5-C2* were more than 94% identical to each other at the nucleotide level. Based on our results and the sequence information of *Cb5* ORFs, members of the two gene pairs *GmCb5-A1* and *GmCb5-A2* and *GmCb5-C3* and *GmCb5-C4* were more than ∼94% identical to each other at the nucleotide level. However, the overall nucleotide identity between the sets of gene pairs *GmCb5-C1* and *-C2* and *GmCb5-C3* and *-C4* were approximately 63%. Since our objective was to assess the role of different classes of Cb5 isoforms, *GmCb5-A2* and *GmCb5-C4* were not cloned.

**Figure 2:**
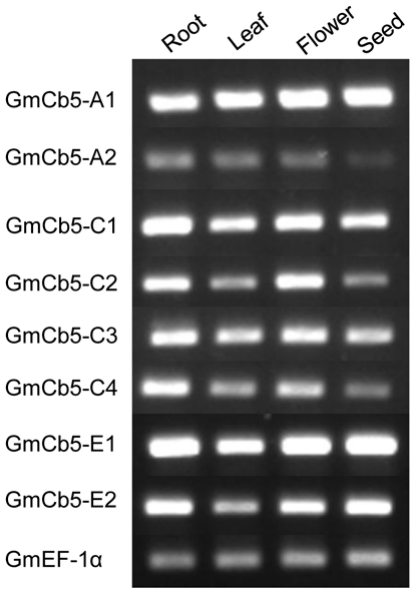
Organ-specific *Cb5* gene expression analyses of soybean. Equal quantities of total RNA from each organ or tissue were analyzed for expression of each *Cb5* gene by semi-quantitative RT-PCR using gene-specific primer pairs. The Ef-1α gene was used as a positive control.

The apparent complexity of the soybean *Cb5* gene family prompted expression profiling with semi-quantitative RT-PCR using gene-specific primer sets (Table S1) to determine transcript abundance of eight *GmCb5* genes in different organs, namely roots, leaves, flowers and immature seeds (R5 stage). All *GmCb5* genes displayed a constitutive expression pattern (Figure 2). However, the display of differential transcript pattern between members of each GmCb5 gene pairs i.e. *GmCb5-A1* and *GmCb5-A2*, *GmCb5-C1* and *GmCb5-C2*, *GmCb5-C3* and *GmCb5-C4* and *GmCb5-E1* and *GmCb52-E2* suggested that the likely paralogs of each gene pair might have different upstream regulation. This occurrence of eight ER-predicted Cb5 proteins in soybean (Figure 1) compared to three in tung [18] and four in *Arabidopsis* [31], was not surprising given the polyploid nature of the soybean genome [33].

### Functional characterization of soybean *Cb5* genes in yeast

**Figure 3:**
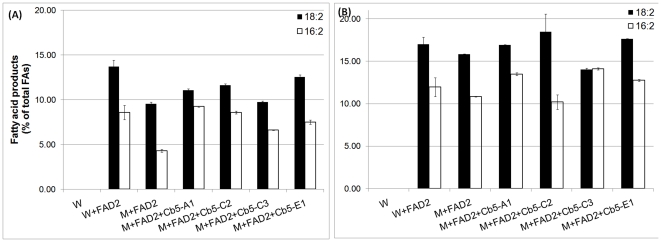
The 16∶2 and 18∶2 content of yeast co-expressing *Cb5* and *FAD2* of soybean. Cultures were induced with galactose and allowed to grow 28°C for 48 h (A) or at 15°C for 96 h (B). FAMES were analyzed by GC-FID. Values are expressed as molar percentage of total FAs and represent average and SD of three independent cultures. W = wild type yeast (empty vector pESC control); M = mutant *cb5* yeast. See Table S2 for detailed fatty acid composition.

Yeast is an excellent experimental system to study fatty acid (FA) desaturation, as it provides a eukaryotic ER, Cb5, and NADH: cytochrome b5 reductase [24]. Moreover, the FA profile of yeast cells lacks PUFAs typically found in plant oils [25], and can easily take up exogenously supplied FA substrates. In order to determine whether cDNA-encoded proteins of soybean *Cb5* ORFs were capable of FA desaturation, the *Cb5* ORFs were co-expressed individually with native *FAD2* (*FAD2-1B*) [34] in a *cb5* deletion mutant of yeast. The total level of di-unsaturated FAs (18∶2 and 16∶2) in mutant *cb5* yeast was less than the wild type (Figure 3A), although considerable levels were still detected, possibly due to interaction of FAD2 with the functional Cb5 domain of stearoyl CoA desaturase [35]. In mutant *cb5* yeast co-expressing *Cb5* genes and *FAD2*, modest increases in total di-unsaturated FA levels were detected compared to those expressing *FAD2* only. This unequivocally demonstrated functionality of the Cb5 proteins in complementing the defects of mutant *cb5* yeast. However, the differences in total di-unsaturated FA levels among four different Cb5 isoforms were not significant, which suggested relatively similar ω-6 desaturation efficiencies. The above result was similar to a previous study, in which no major difference in ω-6 desaturation product levels was observed among Cb5 isoforms of tung expressed in yeast [18].

In the heterologous yeast expression system, the enhanced production of unsaturated FAs at a lower temperature has been demonstrated for many higher plant desaturases, such as FAD2 of *Arabidopsis* [23] and soybean [34] and FAD3 of *Brassica napus* [25]. Consistent with the above findings, a ∼1.5-fold increase in total di-unsaturated FA levels were observed at reduced temperature (Figure 3B), but differences in total di-unsaturated FA levels between the Cb5 isoforms were non-significant. The relatively similar levels of di-unsaturated FA accumulation shown by members of each pair, viz. Cb5-C1 & Cb5-C2 and Cb5-E1 & Cb5-E2 (data not shown), was not unexpected given the significantly high amino acid identity between protein sequences of each pair.

Higher plant FAD2 is capable of desaturating both palmitoleic acid (16∶1) as well as oleic acid (18∶1) [23], [36]. In wild type yeast, which has nearly similar levels of both monounsaturated fatty acids, *Arabidopsis* FAD2 produced roughly 7.5 times as much 18∶2 as 16∶2 [3]. In wild type yeast expressing soybean *FAD2*, we observed ∼1.5 times as much18∶2 as 16∶2 (Figure 3), indicating that soybean FAD2 has a broader substrate preference.

### *Arabidopsis* Cb5 isoforms exhibits differential ω-6 desaturation

**Figure 4:**
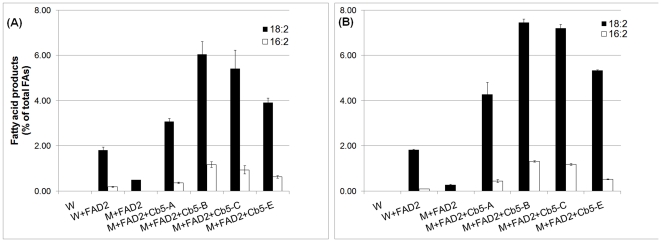
The 16∶2 and 18∶2 content of yeast co-expressing *Cb5 and FAD2* of *Arabidopsis*. Conditions same as described in Figure 3. W = wild type yeast (empty vector pESC control); M = mutant *cb5* yeast. See Table S3 for detailed fatty acid composition.

*Arabidopsis* has six annotated *Cb5* genes in the TAIR database (www.tair.org) and four that are reported to localize to the ER, namely *Cb5-A* (At5g53560), *Cb5-B* (At5g48810), *Cb5-C* (At2g46650) and *Cb5-E* (At2g32720) [17], [21], [31], [37]. *Cb5-D* (At1g26340) is targeted to the outer mitochondrial membrane [31] and *Cb5-F* (At1g60660) is quite distinct in having a predicted transmembrane domain at its N-terminal, as opposed to at the C-terminal as typical with Cb5 proteins [8]. The *Arabidopsis Cb5* genes have been implicated in ER-based FA desaturation [14] but prior to this report their contribution in FA desaturation was not determined. The *Arabidopsis Cb5* genes produced significant levels of di-unsaturated FAs (18∶2 & 16∶2) in mutant *cb5* yeast co-expressing both *Cb5* and *FAD2* (At3g12120) genes, as opposed to those expressing *FAD2* only (Figure 4). However, in contrast to soybean Cb5 isoforms, we observed significant variation in di-unsaturated FA levels among the four Cb5 isoforms of *Arabidopsis*. Both Cb5-B and Cb5-C were able to accumulate ∼1.5- to 2-fold more di-unsaturated FAs than Cb5-E and Cb5-A, respectively (Figure 4A). Despite enhancement of ω-6 desaturation product levels at lower temperature, both Cb5-B and Cb5-C were still able to accumulate ∼1.5-fold more di-unsaturated FAs than Cb5-E and Cb5-A (Figure 4B).

In the heterologous expression system, the products of desaturation depend on several factors such as growth conditions, temperature, substrate availability [23], yeast strain, and concentration of galactose [38]. In our study, the expression of *Arabidopsis FAD2* in wild type yeast consistently showed ∼7 times as much 18∶2 FA as 16∶2 FA, but the overall level of di-unsaturated FAs was 2% of the total FAs, as opposed to the 20–40% reported in a previous study [38]. The above discrepancy could possibly be due to differences in yeast strain, growth conditions, expression vector, or concentration of galactose in the media.

### Cb5 isoforms of soybean and *Arabidopsis* modulate ω-3 desaturation

**Figure 5:**
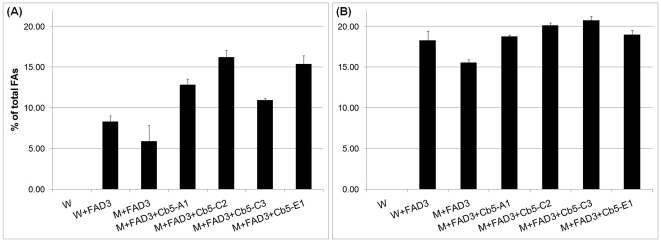
The 18∶3 content of yeast co-expressing *Cb5* and *FAD3* of soybean. A known amount of 18∶2 was added to cultures before induction with galactose and allowed to grow 28°C for 48 h (A) or 15°C for 96 h (B). FAMES were analyzed by GC-FID. Values are expressed as molar percentage of total FAs and represent average and SD of three independent cultures. W = wild type yeast (empty vector pESC control); M = mutant *cb5* yeast. See Table S2 for detailed fatty acid composition.

In the majority of angiosperms, FAD3 is mainly responsible for ω-3 FA production, primarily 18∶3. Accordingly, the contributions of various Cb5 isoforms in ω-3 desaturation were explored using the soybean, *FAD3-1A* [39], which is similar to soybean, *FAD3B* [40]. As expected, in wild type or mutant *cb5* yeast transformed with *FAD3* and not supplemented with exogenous 18∶2, we failed to detect 18∶3 (Table S2). However, under conditions of 18∶2 feeding, considerable production of 18∶3 was observed in both wild type and mutant *cb5* yeast (Figure 5A). Notably, co-expression of soybean native *Cb5* and *FAD3* genes produced a 2-fold more 18∶3 FAs than expressing *FAD3* alone (Figure 5A). While all Cb5 isoforms complemented the defect of *cb5* yeast mutant, there was interesting variation in 18∶3 FA accumulation. Although 18∶3 levels were relatively similar in Cb5-C2 and Cb5-E1, both showed ∼1.5-fold more 18∶3 accumulation than Cb5-C3. In Cb5-A1 the 18∶3 level was intermediate to that of Cb5-C2, Cb5-E1 and Cb5-C. At reduced temperature, the levels of ω-3 desaturation product were higher all around, but there was no distinct variation in 18∶3 levels between the Cb5 isoforms (Figure 5B).

**Figure 6:**
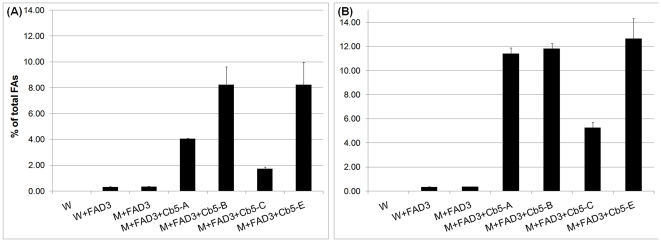
The 18∶3 content of yeast co-expressing *Cb5* and *FAD3* of *Arabidopsis*. Conditions same as described in Figure 5. W = wild type yeast (empty vector pESC control); M = mutant *cb5* yeast. See Table S3 for detailed fatty acid composition.

Similar to soybean, distinct variation in 18∶3 levels between the *Arabidopsis* Cb5 isoforms was observed when the native *Cb5* and *FAD3* (*At2g29980*) genes were co-expressed in mutant *cb5* yeast (Figure 6A). Both Cb5-B and Cb5-E were able to accumulate 2- to 4-fold more 18∶3 than Cb5-A and Cb5-C, respectively. At reduced temperature, both Cb5-B & Cb5-E still yielded ∼2-fold higher 18∶3 level than Cb5-C, while the 18∶3 level in Cb5-A became relatively similar to both Cb5-B & Cb5-E (Figure 6B).

Higher plant FAD3 proteins possess the minor activity to desaturate mono-unsaturated FAs (16∶1 and 18∶1). In a previous study the expression of *Brassica napus FAD3* in wild type yeast was shown to accumulate minor but distinct amounts of 16∶2 (Δ9, 13) and 18∶2 (Δ9, 15) FAs [24]. In our study, with soybean *FAD3* expression in both wild type and mutant *cb5* yeast, similar minor 16∶2 and 18∶2 FA accumulation was observed (Table S2), but their retention times corresponded to those of 16∶2 (Δ9, 12) and 18∶2 (Δ9, 12). These two FAs were completely absent in control samples of both wild type and mutant *cb5* yeast transformed with empty vector. Under the condition of exogenous 18∶2 feeding, the 16∶2 peak appeared consistently in both wild type and mutant *cb5* yeast either expressing soybean *FAD3* alone or co-expressing *FAD3* with either native (Table S2) or non-native (Table S4) *Cb5* genes. Such Δ12 desaturase activity was completely absent with *Arabidopsis* FAD3 (see below). The display of an additional but low level of Δ12 desaturase activity by soybean FAD3, as opposed to ω-3 desaturase activity reported for *Brassica napus* FAD3, could possibly be due to FAD3 originating from two divergent species. It is interesting to note that such bifunctional Δ12/ω-3 desaturase activity have previously been reported for fungus desaturases [41].

### Retention of differential ω-3 desaturation properties by Cb5 isoforms

**Figure 7:**
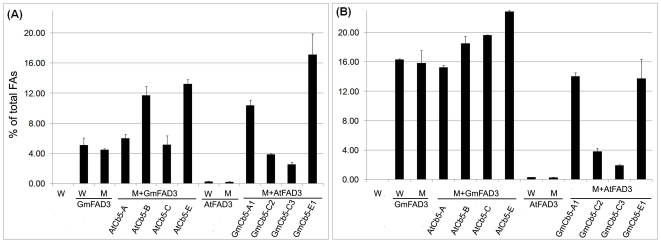
The 18∶3 content of yeast co-expressing *Cb5* of *Arabidopsis* or soybean and non-native *FAD3*. Conditions same as described in Fig. 5. W = wild type yeast (empty vector pESC control); M = mutant *cb5* yeast; Gm = *Glycine max*; At = *Arabidopsis thaliana*. See Table S4 for detailed fatty acid composition.

With the demonstration of differential 18∶3 accumulation by Cb5 isoforms of both soybean and *Arabidopsis* (Figure 5A and 6A), the production profiles of the Cb5 isoforms when co-expressed with non-native *FAD3* was analyzed. For above, soybean *Cb5* (*GmCb5*) was co-expressed with *Arabidopsis FAD3* (*AtFAD3*) and *Arabidopsis Cb5* (*AtCb5*) was co-expressed with soybean *FAD3* (*GmFAD3*) in mutant *cb5* yeast. AtCb5-B and AtCb5-E accumulated ∼3-fold more 18∶3 than both AtCb5-C and AtCb5-A (Figure 7A), reflective of their higher ω-3 desaturation efficiencies as observed with their native FAD3 (Figure 6A). At the lower temperature, both AtCb5-E and AtCb5-B were still able to accumulate comparatively more 18∶3 than AtCb5-A, but the 18∶3 level of AtCb5-C was more similar to AtCb5-B (Figure 7B).

The similarly higher ω-3 desaturation efficiency associated with the soybean Cb5 isoforms GmCb5-A1 and GmCb5-E1 (Figure 5A) was also observed when co-expressed with non-native AtFAD3 (Figure 7A&B). However, there was a significant 3- to 4-fold reduction in 18∶3 accumulation by GmCb5-C2 and GmCb5-C3 compared to those observed with native FAD3 (Figure 5). The *GmCb5* & *GmFAD3* (Figure 5) and *GmCb5* & *AtFAD3* (Figure 7) co-expression experiments were performed independently. Despite the considerable increase in precursor (18∶2) in the latter experiment (compare Tables S2 and S4), the inability to achieve higher 18∶3 accumulation by co-expression of GmCb5-C2 and GmCb5-C3 suggested they interact less efficiently with the non-native AtFAD3.

## Discussion

Two electron transport chains occur in the ER: NADPH: cytochrome P450 reductase with P450s and NADH:cytochrome b5 reductase with Cb5 [17], with the latter implicated as the major route for providing reducing equivalents to ER-based FAD2 (18∶1 to 18∶2 desaturation) and FAD3 (18∶2 to 18∶3 desaturation) desaturases [13], [14]. Biochemical evidence also indicates that both FAD2 and FAD3 of higher plants utilize Cb5 as an electron donor [42]. Here we report the contributions of various Cb5 isoforms from both *Arabidopsis* and soybean to FAD2- and FAD3-mediated reactions using a mutant yeast strain deficient in endogenous *Cb5* gene.

Yeast is a convenient system to study FA desaturation, as it provides a eukaryotic ER and the enzymes Cb5 and NADH: cytochrome b5 reductase. Several factors associated with yeast metabolism, such as the rate of FA synthesis, rate of FA breakdown and rate of exogenous FA incorporation, determine the level of final product accumulation, but the data are considered semi-quantitative [24]. In the present study, we observed that different Cb5 isoforms of *Arabidopsis* were not similarly efficient in ω-6 desaturation, as evidenced by display of ∼1.5- to 2-fold higher di-unsaturated FA (18∶2 and 16∶2) accumulation by both Cb5-B and Cb5-C at both higher (Figure 4A) and lower temperature (Figure 4B). In the case of soybean Cb5 isoforms, the differences in total di-unsaturated FA levels at both higher (Figure 3A) and lower (Figure 3B) temperatures were not significant. Both soybean and *Arabidopsis* experiments were performed independently, but FAD2 precursors (18∶1 and 16∶1) were similarly available under comparable growth conditions (Tables S2 and S3). Moreover, as *FAD2* and *Cb5* of both soybean and *Arabidopsis* were driven by the same *GAL10* and *GAL1* promoters, respectively (see methods), it is not likely that differential transcript levels contributed to variable ω-6 desaturation displayed by *Arabidopsis* or non-variable desaturation demonstrated by soybean Cb5 isoforms.

During our investigation we consistently observed a ∼10-fold higher di-unsaturated FA accumulation in mutant *cb5* yeast expressing soybean *FAD2* as compared to *Arabidopsis FAD2*, despite the availability of relatively similar level of precursors (compare Tables S2 and S3). Additionally, in contrast to *Arabidopsis*, the difference in desaturation products, particularly 18∶2 levels, between mutant *cb5* yeast expressing either *FAD2* alone or co-expressing both *Cb5* and *FAD2* of soybean were less. This suggested that majority of ω-6 desaturation product in mutant *cb5* yeast is being contributed by plausible interaction of functional Cb5 domain of endogenous stearoyl CoA desaturase [35] with soybean FAD2. Such preferential interaction of Cb5 domain of endogenous stearoyl CoA desaturase with soybean FAD2 or vice-versa in ω-6 desaturation is subject of future investigation but it certainly precluded our effort in assessing the individual contribution of soybean Cb5 isoforms in ω-6 desaturation. Taken together, it would be interesting to explore the contribution of soybean Cb5 isoforms in ω-6 desaturation by using an *ole1 cb5* double mutant of yeast, deficient in both stearoyl CoA desaturase and microsomal *Cb5*.

The Cb5 isoforms of both soybean and *Arabidopsis* showed significant variation in ω-3 desaturation when expressed with their native FAD3 under comparable growth conditions. The soybean Cb5-C2, Cb5-E1 and Cb5-A1 (Figure 5A) and *Arabidopsis* Cb5-B and Cb5-E (Figure 6A) consistently showed higher 18∶3 accumulation compared to the other isoforms. Most importantly, such differential properties associated with most Cb5 isoforms of soybean and *Arabidopsis* were consistently observed with the non-native FAD3 also (Figure 7). Additionally, as *FAD3* and *Cb5* were driven by the same *GAL10* and *GAL1* promoters, respectively (see methods), the differential transcript accumulation is not likely the cause of the apparent differential behaviour of Cb5 isoforms in ω-3 desaturation.

Among ER-localized proteins, fatty acid desaturases (FADs) have a much shorter half-life. For example, mammalian stearoyl-CoA desaturase, which is structurally related to plant FAD2/FAD3 enzymes, has a half-life of 4 hours compared to the 2–6 day half-life of ER-resident Cb5 proteins ([43] and references therein). The half-life of ER-resident FAD proteins can be modulated by environmental factors. For example, the enhancement in unsaturation at cold temperature by higher plant FAD2 [34] and FAD3 [25] in the heterologous yeast system is correlated to the increase in the steady state level of the desaturase. Taking this into consideration, it seems probable that an increase in the abundance of either the soybean (Figure 5B) or *Arabidopsis* (Figure 6B) FAD3 proteins resulted in non-discrimination in 18∶3 levels at lower temperature condition between most of the Cb5 isoforms.

Although retention of differential ω-3 desaturation activity was observed for most of the soybean and *Arabidopsis* Cb5 isoforms , the significant ∼3-fold reductions in 18∶3 level with *Gm*Cb5-C2 and *Gm*Cb5-C3, particularly with non-native AtFAD3 (Figure 7), were intriguing. It is important to note that *Arabidopsis Cb5-C* accumulated ∼2- to 4-fold less 18∶3 than other Cb5 proteins co-expressed with the corresponding native *FAD3* (Figure 6). On the contrary, co-expression of *AtCb5-C* with non-native *GmFAD3* resulted in significant 2- and 4-fold increases in 18∶3 level under standard and lower temperature conditions, respectively, (Figures 7A and B) compared to the levels during co-expression with native *FAD3*, (Figures 6A and B). In general, the observation of higher 18∶3 accumulation levels with the ‘C’ class of Cb5 proteins, whether from soybean or *Arabidopsis*, with soybean FAD3 suggested better interaction efficiencies of “C” class Cb5 proteins with soybean FAD3.

In phylogenetic analyses, among four arbitrary classes of Cb5 proteins (A, B, C and E; Figure 1), the ‘C’ class from soybean formed a separate group that clustered close to *At*Cb5-C. The amino acid alignment of different Cb5 proteins from soybean, Tung and *Arabidopsis* indicated that, despite significantly higher level of overall identity of ‘C’ class proteins with other classes, there was considerable diversity at the C-termini (boxed), particularly in the region preceding the trans-membrane domain (Figure S1). Whether such differences in amino acids are of any significance for greater interaction efficiency of ‘C’ class Cb5 proteins with soybean FAD3 as opposed to *Arabidopsis* FAD3 is open for future investigations, but our results indicate that evolutionary divergence of Cb5 isoforms might have contributed to their differential interaction efficiencies with desaturases causing variations in product outcome of fatty acid desaturation. Such information indicates the feasibility to modulate FA desaturation in the targeted organisms through genetic engineering approaches.

## Materials and Methods

### Cloning and plasmid construction

Total RNA from leaf and seed tissues of soybean (*Glycine max* var. Williams 82) was isolated using TRIZOL reagent (Invitrogen, USA). Total RNA was isolated from *Arabidopsis* leaf tissue by RNAqueous kit (Ambion, cat# 1912). The RNA was treated with DNaseI (Turbo DNA-free DNaseI, Ambion) according to the manufacturer's instructions to remove genomic DNA contamination. The concentration of DNaseI-treated RNA was determined with the NanoDrop ND-1000 UV-Vis spectrophotometer (NanoDrop Technologies, Wilmington, DE, USA). RNA was used to generate cDNA pool using oligo (dT)_12–18_ and Superscript III reverse transcriptase (Invitrogen, USA) by following manufacturer's protocol. To simplify subsequent directional cloning, the primers for soybean *Cb5* genes included the restriction sites *Bam*HI/*Sal*I at the 5′- and *Hind*III at the 3′- end, respectively, and for *FAD2* & *FAD3* contained *Cla*I and *Sac*I at the 5′- and 3′-end, respectively. Similarly, primers for *Arabidopsis Cb5* included 5′ *Bam*HI and 3′ *Hind*III and for *Arabidopsis* FAD2 & FAD3 included *Not*I and *Pac*I at the 5′- and 3′-end, respectively (Table S5). The cDNAs of the respective genes were amplified by KOD hot start DNA polymerase (Novagen, USA) per manufacturer's protocol by using gene-specific primers. The cDNAs were initially cloned in pKS(−) at EcoRV site, and their sequences were verified by bidirectional sequencing. From sequence-verified clones, the cDNA inserts were subsequently excised and directionally cloned into the yeast expression vector pESc-URA (Stratagene, USA). Plasmids were constructed by standard molecular biology techniques [44]. The *Cb5* genes of both soybean and *Arabidopsis* were cloned behind the galactose-inducible *GAL1* promoter whereas *FAD2/FAD3* genes were placed behind the galactose-inducible *GAL10* promoter.

### Semi-quantitative RT-PCR

Organ-specific expression patterns for each soybean *Cb5* gene were analyzed by semi-quantitative RT-PCR. The DNaseI-digested RNA of seeds (early R5), roots, young leaves and flowers were quantified by Nano-drop, and equal quantities of RNA were used as template for RT-PCR reaction, essentially as described above. The product of RT was diluted with equal volume of water and 4 µL of each diluted reaction was used as template in a 20-µL PCR reaction containing gene-specific primers (Table S1). The amplification conditions were as follows: 94°C for 3 min, and 29 cycles of 94°C for 30 s, 55°C for 30 s, and 72°C for 1 min. Following PCR, the products were diluted further with 10 µL of water and loading dye. One-third volume of each diluted reaction was analyzed by agarose gel electrophoresis. The degree of gene expression correlated to the relative intensity of each band as determined by visual comparison of the ethidium bromide staining intensity. The elongation factor Ef-1α gene [45] was used as positive control.

### Yeast Strains, transformation and culture conditions

The yeast strains used in this study included *Saccharomyces cerevisiae* Hansen (*matα his3 leu2 lys2 ura3*; cat# 95400. BY4742, Invitrogen) and the mutant strain that carries a disruption in the microsomal Cb5 gene (cat# 95400.17382) in wild-type BY4742. Yeast cells were transformed by Fast-Yeast Transformation kit (G-Bioscience, MO, USA) according to manufacturer's protocol. Transformants were selected from minimal SD agar (Clontech, cat# 630412) plate supplemented with appropriate auxotrophic supplements (-URA DO mixture, Clontech, cat# 630416) maintained at 30°C. The identities of different yeast transformants were verified using gene-specific primers. Individual colonies of transformed cells were then grown for 2 days at 30°C in minimal SD media (Clontech, cat# 630411) lacking uracil to generate preculture (OD_600_∼1.4–1.5). The amount of preculture necessary to obtain an OD_600_ of 0.2 in 6 mL of induction media (SD Gal/Raf, Clontech, cat# 630420) was pelleted at 1800× g for 5 minutes at +4°C. The pellets were washed once with 3 mL of induction media. The washed pellets were finally resuspended in 6 mL of induction media and incubated either at 28°C for 48 h or 15°C for 96 h with shaking at 250 rpm. Linoleic acid (Nu-Chek Prep, Elysian, MN) when included, were added to the induction medium at a final concentration of 0.05% (v/v) along with 0.2% tergitol type NP-40 (Sigma) in order to solubilize the fatty acids.

### Fatty acid analyses

To analyze fatty acids, 5 mL of induced yeast cultures (OD_600_∼1.4–1.5) were harvested by centrifugation. The pellets were subsequently washed once with 3 mL of water and dried under vacuum. Cultures supplemented with exogenous FAs were washed first with 3 mL of 0.2% (v/v) tergitol type NP-40 (Sigma) and then twice with water. FA analyses were performed as described [13]. Briefly, to the washed pellets 1 mL of 2.5% sulfuric acid (v/v) in methanol and a known amount of 17∶0 as internal standard were added and incubated at 80°C for an hour. After cooling, FAMES (fatty acid methyl esters) were recovered in 200 µL of hexane, and 1 µL was injected (split ratio 10∶1) into a GC (Trace GC ultra, Thermo Electron Corporation) fitted with a 30-m×0.25-mm (i.d.) TR-FAME column (Thermo Electron Corporation) and quantified using a flame ionization detector. The GC oven temperature was programmed to hold at 150°C for 1 min, increase to 180°C at 10°C per min, and then hold for an additional 6 min. Peak identifications were confirmed with commercially available methyl esters of standards (Nu-Chek Prep, Elysian, MN). All analyses were performed on triplicate samples and replicated three times.

### Phylogenetic analysis

The amino acid sequences of predicted Cb5 proteins were aligned using ClustalX [46] with the default parameters (gap open penalty = 10, gap extension penalty = 0.2). The unrooted phylogenetic tree was constructed by the neighbor-joining method using MEGA4 software [47]. The confidence level of monophyletic groups was estimated using a bootstrap analysis of 1000 replicates.

## Supporting Information

Figure S1 **Deduced amino acid sequence alignment of Cb5 proteins.**
(PDF)

Table S1 **Primers of soybean** ***Cb5*** **(** ***GmCb5*** **) genes used for RT-PCR analyses.**
(PDF)

Table S2 **Fatty acid composition of yeast co-expressing** ***Cb5 and FAD2/FAD3*** **of soybean.**
(PDF)

Table S3 **Fatty acid composition of yeast co-expressing** ***Cb5 and FAD2/FAD3*** **of Arabidopsis.**
(PDF)

Table S4 **Fatty acid composition of yeast co-expressing** ***Cb5*** **of Arabidopsis (A) or soybean (B) and non-native** ***FAD3*** **.**
(PDF)

Table S5 **Primer used for cloning of** ***Cb5*** **,** ***FAD2*** **and** ***FAD3*** **of soybean (A) and Arabidopsis (B)**.
(PDF)
