# Supplementary material for: Higher Plant Cytochrome b5 Polypeptides Modulate Fatty Acid Desaturation
Source: PLoS One. 2012 Feb 23;7(2):e31370. doi: 10.1371/journal.pone.0031370 (PMC3285619; doi:10.1371/journal.pone.0031370)
Supplement: Figure S1 — Deduced amino acid sequence alignment of Cb5 proteins. (PDF) [file pone.0031370.s001.pdf]

Figure S1. Deduced amino acid sequence alignment of Cb5 proteins

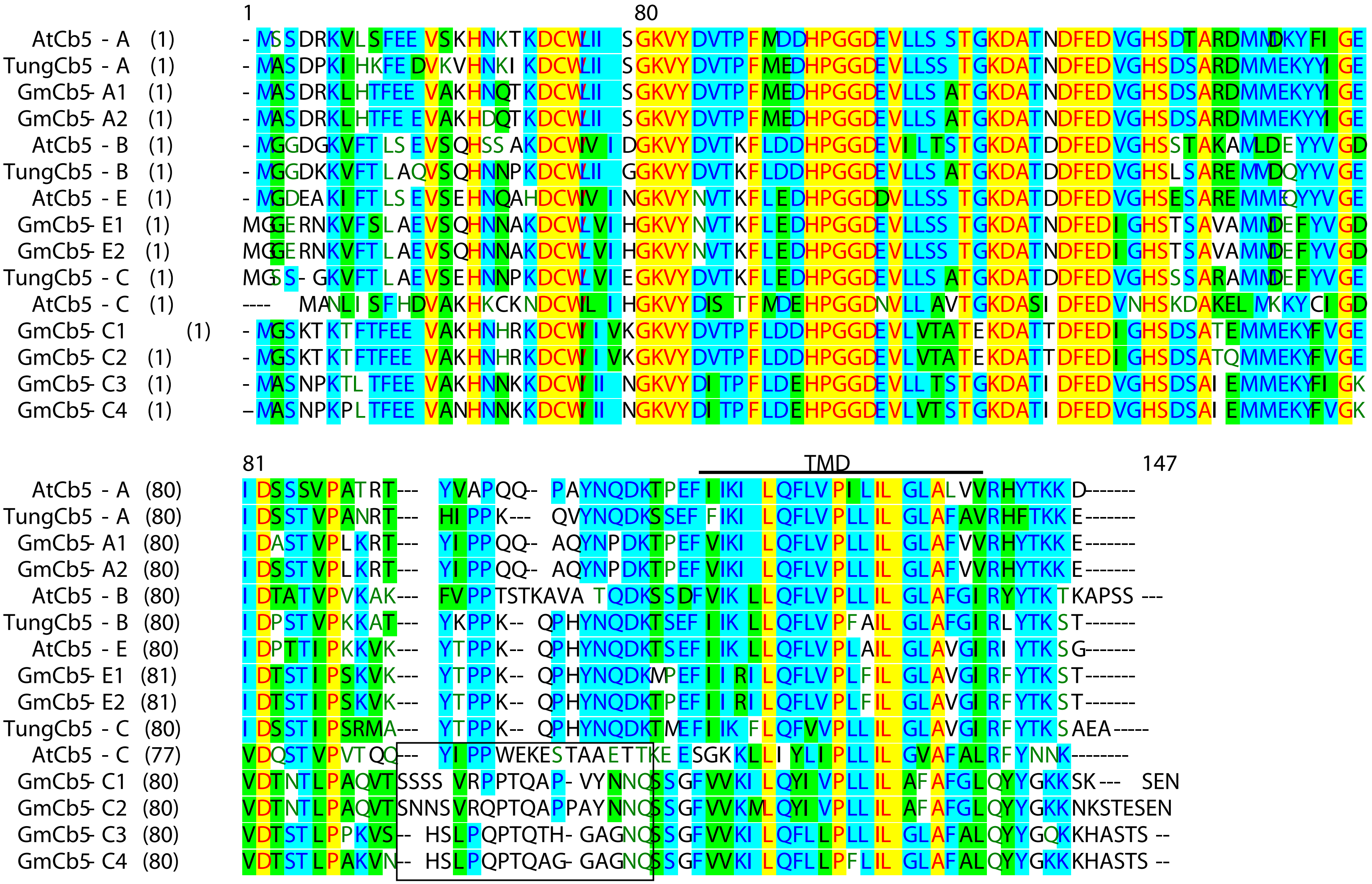

The deduced amino acid sequence of soybean Cb5 (GmCb5) isoforms were aligned with known and putative ER targeted Cb5 proteins of Tung and Arabidopsis using vector NTI software. Accession number of soybean Cb5 is provided in Table1. Accession number of Arabidopsis Cb5: AtCb5-A (At5g53560), AtCb5-B (At5g48810), AtCb5-C (At2g46650) and AtCb5-E (At2g32720). Accession number of Tung Cb5: TungCb5-A (AY578727), TungCb5-B (AY578728) and TungCb5-C (AY578729). The depiction of transmembrane domain (TMD) is based on an earlier report [18].
