# Supplementary material for: Higher Plant Cytochrome b5 Polypeptides Modulate Fatty Acid Desaturation
Source: PLoS One. 2012 Feb 23;7(2):e31370. doi: 10.1371/journal.pone.0031370 (PMC3285619; doi:10.1371/journal.pone.0031370)
Supplement: Table S1 — Primers of soybean Cb5 ( GmCb5 ) genes used for RT-PCR analyses. (PDF) [file pone.0031370.s002.pdf]

**Table S1. Primers of soybean Cb5 (GmCb5) genes used for RT-PCR analyses**

| Genes | Forward | Reverse |
|---|---|---|
| GmCb5-A1 | TCGGAAACTTCACACTTTT | TCACAAATTCTGGAGTCTTA |
| GmCb5-A2 | TCGGAAACTTCACACTTTC | TCACAAATTCTGGAGTCTTG |
| GmCb5-C1 | ATGGGTTCAAAAACCAAGAC | TCAATTTTCTGACTTGC |
| GmCb5-C2 | same as above | TCAATTTTCTGACTCAGTG |
| GmCb5-C3 | CTTTGAGGAGGTAGCTAAG | TGCGAGGCCCAGTATCAAC |
| GmCb5-C4 | TTTTGAGGAGGTGGCTAAT | GGCAAGGCCCAATATCAAG |
| GmCb5-E1 | ATGGGTGGGGAGCGGAAC | TTATGTTGATTTGGTGTAGAAAC |
| GmCb5-E2 | ATGGGTGGGGAGCGC | TCATGTTGATTTGGTGTAGAAACGAATG |
| Ef-1α | CAGACTCGTGAACATGCTCTGC | TACCTGGCCTTGGAATACTTGG |
