# Supplementary material for: Higher Plant Cytochrome b5 Polypeptides Modulate Fatty Acid Desaturation
Source: PLoS One. 2012 Feb 23;7(2):e31370. doi: 10.1371/journal.pone.0031370 (PMC3285619; doi:10.1371/journal.pone.0031370)
Supplement: Table S2 — Fatty acid composition of yeast co-expressing Cb5 and FAD2/FAD3 of soybean. (PDF) [file pone.0031370.s003.pdf]

Table S2. Fatty acids composition of yeast co-expressing Cb5 and FAD2/FAD3 of soybean

	16:0		16:1		16:2		18:0		18:1		18:2		18:3	
	28^{0}C	15^{0}C	28^{0}C	15^{0}C	28^{0}C	15^{0}C	28^{0}C	15^{0}C	28^{0}C	15^{0}C	28^{0}C	15^{0}C	28^{0}C	15^{0}C
W(empty pESC)	23.9 ± 0.19	21.9 ± 0.09	38.2 ± 0.30	44.9 ± 0.05			9.6 ± 0.14	6.6 ± 0.11	28.3 ± 0.44	26.5 ± 0.25				
M(empty pESC)	24.2 ± 0.27	21.1 ± 0.39	39.0 ± 0.40	45.2 ± 0.30			9.2 ± 0.10	6.6 ± 0.01	27.6 ± 0.37	27.1 ± 0.11				
W+FAD2	21.6 ± 0.07	19.0 ± 0.21	26.4 ± 1.02	27.6 ± 1.11	8.6 ± 0.80	11.9 ± 1.10	12.1± 0.10	8.6 ± 0.09	17.7 ± 0.54	16.0 ± 0.66	13.7 ± 0.73	17.0 ± 0.85		
M+FAD2	22.6 ± 0.12	18.7 ± 0.06	29.1 ± 0.21	30.1 ± 0.13	4.3 ± 0.15	10.8 ± 0.03	11.3 ± 0.03	8.1 ± 0.05	23.2 ± 0.12	16.5 ± 0.02	9.5 ± 0.21	15.8 ± 0.05		
M+FAD2+Cb5-A1	20.4 ± 0.02	18.9 ± 0.05	29.7 ± 0.18	26.8 ± 0.05	9.2 ± 0.07	13.5 ± 0.16	8.9 ± 0.04	8.3 ± 0.04	20.8 ± 0.09	15.7 ± 0.07	11.1 ± 0.17	16.9 ± 0.04		
M+FAD2+Cb5-C2	22.9 ± 0.05	14.9 ± 1.87	27.1 ± 0.34	25.2 ± 1.79	8.6 ± 0.18	10.2 ± 0.86	11.3 ± 0.07	10.7 ± 0.14	18.6 ± 0.12	20.6 ± 0.83	11.6 ± 0.17	18.4 ± 2.12		
M+FAD2+Cb5-C3	20.6 ± 0.57	20.5 ± 0.07	26.6 ± 0.21	27.5 ± 0.12	6.6 ± 0.03	14.1 ± 0.13	13.6 ± 0.08	8.6 ± 0.11	23.0 ± 0.06	15.3 ± 0.05	9.7 ± 0.12	14.0 ± 0.15		
M+FAD2+Cb5-E1	22.4 ± 0.12	18.8 ± 0.04	26.7 ± 0.41	26.2 ± 0.08	7.5 ± 0.23	12.7 ± 0.10	11.4 ± 0.11	8.5 ± 0.05	19.5 ± 0.28	16.2 ± 0.03	12.5 ± 0.25	17.6 ± 0.06		
W+FAD3*	22.8 ± 0.12	20.5 ± 0.09	36.0 ± 0.02	42.7 ± 0.06	1.0 ± 0.05	1.6 ± 0.04	10.6 ± 0.08	6.9 ± 0.04	29.1 ± 0.13	27.6 ± 0.24	0.5 ± 0.01	0.8 ± 0.02		
M+FAD3*	24.0 ± 0.04	17.0 ± 0.19	33.5 ± 0.08	41.4 ± 0.21	0.7 ± 0.01	1.6 ± 0.01	10.7 ± 0.04	7.4 ± 0.06	30.8 ± 0.15	31.6 ± 0.04	0.4 ± 0.02	1.1 ± 0.01		
W(empty pESC)	23.9 ± 0.85	21.5 ± 0.45	4.2 ± 0.94	8.5 ± 0.45			8.7 ± 0.49	5.2 ± 0.07	3.0 ± 0.72	4.7 ± 0.29	60.3 ± 3.00	60.2 ± 1.25		
M(empty pESC)	24.5 ± 0.59	20.9 ± 0.33	4.2 ± 0.95	8.5 ± 0.48			8.7 ± 0.32	4.8 ± 0.23	2.8 ± 0.69	4.6 ± 0.30	59.8 ± 2.48	61.1 ± 1.18		
W+FAD3	19.9 ± 1.02	19.1 ± 0.38	3.4 ± 0.37	7.4 ± 0.99	0.1 ± 0.01	0.1 ± 0.09	8.2 ± 0.58	5.1 ± 0.26	2.7 ± 0.33	4.7 ± 0.72	57.4 ± 2.97	45.3 ± 2.89	8.3 ± 0.70	18.3 ± 1.17
M+FAD3	16.0 ± 4.63	13.5 ± 0.21	6.1 ± 3.25	12.0 ± 0.28	0.1 ± 0.04	0.3 ± 0.01	6.8 ± 2.09	4.8 ± 0.20	5.7 ± 3.11	9.3 ± 0.27	59.5 ± 15.01	44.5 ± 1.08	5.9 ± 1.92	15.6 ± 0.37
M+FAD3+Cb5-A1	15.6 ± 0.89	16.6 ± 0.24	2.6 ± 0.20	6.0 ± 0.13	0.2 ± 0.01	0.2 ± 0.01	6.7 ± 0.39	4.7 ± 0.11	2.1 ± 0.17	3.9 ± 0.13	60.1 ± 2.36	49.8 ± 0.79	12.8 ± 0.75	18.7 ± 0.21
M+FAD3+Cb5-C2	22.6 ± 0.48	17.8 ± 0.35	4.7 ± 0.57	5.8 ± 0.33	0.3 ± 0.07	0.2 ± 0.01	9.0 ± 0.19	4.7 ± 0.08	3.6 ± 0.39	3.6 ± 0.20	43.5 ± 1.74	47.9 ± 1.29	16.2 ± 0.87	20.1 ± 0.33
M+FAD3+Cb5-C3	20.5 ± 0.42	19.2 ± 0.19	4.3 ± 0.76	6.7 ± 0.15	0.2 ± 0.06	0.2 ± 0.01	10.8 ± 0.16	5.3 ± 0.11	4.1 ± 0.70	4.2 ± 0.14	49.0 ± 2.35	43.7 ± 0.17	10.9 ± 0.24	20.7 ± 0.50
M+FAD3+Cb5-E1	17.4 ± 1.73	16.3 ± 0.15	3.0 ± 0.69	6.1 ± 0.18	0.2 ± 0.05	0.2 ± 0.01	7.0 ± 0.84	4.5 ± 0.06	2.4 ± 0.58	4.1 ± 0.10	54.6 ± 4.89	49.8 ± 1.03	15.4 ± 1.00	19.0 ± 0.56

For Cb5 and FAD3 co-expression study 18:2 were added to the culture before induction with galactose. FAMES were analyzed by GC-FID. Induction time for culture maintained at 28^{0}C and 15^{0}C were 48 and 96 hour respectively. Values represents mol percentage of total fatty acids. ± represents SD of three independent cultures. W= wild type yeast; M= mutant yeast disrupted in endogenous Cb5 gene; ND= Not detected. *Clutures not fed with 18:2.
