# Supplementary material for: Higher Plant Cytochrome b5 Polypeptides Modulate Fatty Acid Desaturation
Source: PLoS One. 2012 Feb 23;7(2):e31370. doi: 10.1371/journal.pone.0031370 (PMC3285619; doi:10.1371/journal.pone.0031370)
Supplement: Table S3 — Fatty acid composition of yeast co-expressing Cb5 and FAD2/FAD3 of Arabidopsis. (PDF) [file pone.0031370.s004.pdf]

Table S3. Fatty acids composition of yeast co-expressing *Cb5* and *FAD2/FAD3* of *Arabidopsis*

Constructs	16:0		16:1		16:2		18:0		18:1		18:2		18:3	
	28^{0}C	15^{0}C	28^{0}C	15^{0}C	28^{0}C	15^{0}C	28^{0}C	15^{0}C	28^{0}C	15^{0}C	28^{0}C	15^{0}C	28^{0}C	15^{0}C
W(empty pESC)	23.9 ± 0.27	21.9 ± 0.09	39.0 ± 0.18	45.8 ± 0.09			9.3 ± 0.13	6.4 ± 0.04	27.8 ± 0.33	25.9 ± 0.16				
M(empty pESC)	23.1 ± 0.08	21.5 ± 0.33	39.1 ± 0.69	46.0 ± 0.72			8.7 ± 0.11	6.1 ± 0.18	29.1 ± 0.65	26.3 ± 0.27				
W+FAD2	23.3 ± 0.22	21.9 ± 0.02	39.1 ± 0.34	45.1 ± 0.01	0.2 ± 0.03	0.1 ± 0.00	9.0 ± 0.14	6.3 ± 0.12	26.6 ± 0.01	24.8 ± 0.15	1.8 ± 0.14	1.8 ± 0.03		
M+FAD2	22.8 ± 0.10	22.0 ± 0.90	39.3 ± 0.28	46.0 ± 0.83	ND	ND	8.7 ± 0.07	6.1 ± 0.27	28.8 ± 0.32	25.7 ± 0.31	0.5 ± 0.01	0.3 ± 0.03		
M+FAD2+Cb5-A	22.4 ± 0.05	19.7 ± 0.25	39.5 ± 0.08	47.5 ± 0.23	0.4 ± 0.03	0.4 ± 0.07	8.8 ± 0.05	5.9 ± 0.19	25.8 ± 0.20	22.2 ± 0.74	3.1 ± 0.16	4.3 ± 0.54		
M+FAD2+Cb5-B	22.8 ± 0.12	22.5 ± 0.09	36.9 ± 0.17	43.1 ± 0.03	1.2 ± 0.13	1.3 ± 0.03	9.2 ± 0.22	6.7 ± 0.17	23.9 ± 0.60	18.9 ± 0.04	6.0 ± 0.58	7.4 ± 0.16		
M+FAD2+Cb5-C	22.9 ± 0.07	21.5 ± 0.03	38.1 ± 0.28	44.1 ± 0.17	0.9 ± 0.19	1.2 ± 0.04	9.0 ± 0.05	6.4 ± 0.07	23.7 ± 0.86	19.6 ± 0.17	5.4 ± 0.84	7.2 ± 0.17		
M+FAD2+Cb5-E	21.8 ± 0.05	18.2 ± 0.13	40.3 ± 0.14	47.7 ± 0.16	0.6 ± 0.06	0.5 ± 0.01	8.8 ± 0.13	6.6 ± 0.07	24.5 ± 0.28	21.6 ± 0.12	3.9 ± 0.22	5.3 ± 0.04		
W+FAD3*	22.8 ± 0.28	21.4 ± 0.17	38.8 ± 0.52	45.7 ± 0.06			9.1 ± 0.30	6.0 ± 0.29	29.3 ± 0.10	26.9 ± 0.40				
M+FAD3*	22.8 ± 0.29	21.2 ± 0.16	38.4 ± 0.21	46.0 ± 0.03			9.1 ± 0.15	6.1 ± 0.16	29.7 ± 0.23	26.7 ± 0.34				
W(empty pESC)	21.9 ± 1.17	21.7 ± 0.13	2.6 ± 0.33	8.4 ± 0.17			7.8 ± 0.43	4.9 ± 0.08	1.8 ± 0.20	4.4 ± 0.11	65.9 ± 2.11	60.6 ± 0.25		
W(empty pESC)	22.9 ± 0.29	21.5 ± 0.53	3.1 ± 0.26	8.7 ± 0.46			8.4 ± 0.14	4.7 ± 0.11	2.1 ± 0.19	4.5 ± 0.27	63.3 ± 0.84	60.6 ± 1.32		
W+FAD3	17.1 ± 2.18	20.7 ± 0.21	1.9 ± 0.41	7.7 ± 0.32			6.0 ± 0.82	4.5 ± 0.07	1.3 ± 0.26	4.1 ± 0.15	73.4 ± 3.73	62.6 ± 0.63	0.3 ± 0.06	0.3 ± 0.01
M+FAD3	17.8 ± 0.76	21.2 ± 0.36	2.0 ± 0.20	8.3 ± 0.29			6.6 ± 0.04	4.8 ± 0.28	1.2 ± 0.10	4.4 ± 0.19	72.1 ± 1.11	61.0 ± 1.09	0.3 ± 0.02	0.3 ± 0.01
M+FAD3+Cb5-A	15.5 ± 0.30	17.6 ± 0.90	2.7 ± 0.10	7.6 ± 0.36			5.9 ± 0.03	4.4 ± 0.48	2.0 ± 0.05	4.6 ± 0.84	69.9 ± 0.46	54.5 ± 2.85	4.0 ± 0.05	11.4 ± 0.49
M+FAD3+Cb5-B	16.9 ± 0.59	19.0 ± 0.37	2.2 ± 0.02	6.1 ± 0.47			6.2 ± 0.26	4.6 ± 0.06	1.4 ± 0.03	3.5 ± 0.22	65.2 ± 1.05	55.1 ± 1.43	8.2 ± 1.40	11.8 ± 0.42
M+FAD3+Cb5-C	19.4 ± 0.88	19.2 ± 0.82	2.6 ± 0.19	6.2 ± 0.57			7.3 ± 0.10	4.7 ± 0.41	1.8 ± 0.15	3.4 ± 0.37	67.2 ± 1.16	61.3 ± 2.63	1.7 ± 0.15	5.2 ± 0.47
M+FAD3+Cb5-E	17.8 ± 0.65	18.7 ± 0.30	2.8 ± 0.11	9.0 ± 0.45			6.9 ± 0.14	5.3 ± 0.21	2.0 ± 0.10	4.7 ± 0.20	62.3 ± 1.13	49.5 ± 2.82	8.2 ± 1.77	12.6 ±1.66

For Cb5 and FAD3 co-expression study 18:2 were added to the culture before induction with galactose. FAMES were analyzed by GC-FID. Induction time for culture maintained at 28^{0}C and 15^{0}C were 48 and 96 hour respectively. Values represents mol percentage of total fatty acids. ± represents SD of three independent cultures. W= wild type yeast; M= mutant yeast disrupted in endogenous Cb5 gene; ND= Not detected. *Clutures not fed with 18:2.
