# Supplementary material for: Higher Plant Cytochrome b5 Polypeptides Modulate Fatty Acid Desaturation
Source: PLoS One. 2012 Feb 23;7(2):e31370. doi: 10.1371/journal.pone.0031370 (PMC3285619; doi:10.1371/journal.pone.0031370)
Supplement: Table S4 — Fatty acid composition of yeast co-expressing Cb5 of Arabidopsis (A) or soybean (B) and non-native FAD3 . (PDF) [file pone.0031370.s005.pdf]

Table S4. **Fatty acids composition of yeast co-expressing *Cb5* of *Arabidopsis* (A) or soybean (B) with non-native *FAD3*, respectively.**

Constructs	16:0		16:1		16:2		18:0		18:1		18:2		18:3	
	28^{0}C	15^{0}C	28^{0}C	15^{0}C	28^{0}C	15^{0}C	28^{0}C	15^{0}C	28^{0}C	15^{0}C	28^{0}C	15^{0}C	28^{0}C	15^{0}C
W(empty pESC)	13.3 ± 0.43	16.7 ± 0.19	1.0 ± 0.05	4.7 ± 0.11			4.6 ± 0.11	3.9 ± 0.05	0.7 ± 0.04	2.6 ± 0.04	80.4 ± 0.62	72.0 ± 0.28		
**A**														
W+GmFAD3	16.4 ± 1.83	15.2 ± 0.16	1.8 ± 0.36	5.6 ± 0.14	ND	0.2 ± 0.00	6.3 ± 0.63	4.2 ± 0.04	1.4 ± 0.27	3.7 ± 0.09	68.8 ± 3.13	54.8 ± 0.34	5.1 ± 0.95	16.3 ± 0.09
M+GmFAD3	14.3 ± 0.58	17.3 ± 2.66	1.5 ± 0.08	5.8 ± 0.50	ND	0.2 ± 0.03	5.5 ± 0.30	4.7 ± 0.76	1.1 ± 0.07	3.9 ± 0.17	73.0 ± 1.16	52.4 ± 1.17	4.4 ± 0.14	15.9 ± 1.67
M+GmFAD3+AtCb5-A	16.4 ± 1.27	17.2 ± 0.27	2.9 ± 0.34	7.4 ± 0.20	0.2 ± 0.02	0.3 ± 0.00	6.2 ± 0.55	4.7 ± 0.14	2.2 ± 0.25	4.6 ± 0.16	66.0 ± 2.94	50.5 ± 1.02	6.0 ± 0.51	15.3 ± 0.27
M+GmFAD3+AtCb5-B	16.8 ± 0.11	17.6 ± 0.83	2.1 ± 0.07	7.9 ± 0.69	0.2 ± 0.03	0.3 ± 0.02	6.7 ± 0.11	4.7 ± 0.36	1.7 ± 0.04	5.4 ± 0.49	60.6 ± 1.35	45.6 ± 3.34	11.7 ± 1.21	18.5 ± 0.96
M+GmFAD3+AtCb5-C	16.1 ± 2.44	17.8 ± 0.06	1.7 ± 0.42	6.3 ± 0.14	0.1 ± 0.02	0.3 ± 0.02	6.3 ± 0.99	4.9 ± 0.05	1.3 ± 0.32	4.2 ± 0.09	69.3 ± 5.35	46.8 ± 0.24	5.2 ± 1.17	19.6 ± 0.06
M+GmFAD3+AtCb5-E	20.0 ± 0.20	19.2 ± 0.16	3.0 ± 0.12	9.9 ± 0.32	0.2 ± 0.01	0.5 ± 0.01	7.8 ± 0.06	5.8 ± 0.08	2.2 ± 0.05	5.8 ± 0.18	53.5 ± 0.96	36.0 ± 0.84	13.3 ± 0.56	22.9 ± 0.12
**B**														
W+AtFAD3	13.5 ± 0.68	17.6 ± 1.09	1.1 ± 0.06	5.4 ± 0.60			4.7 ± 0.33	4.3 ± 0.31	0.8 ± 0.07	3.0 ± 0.35	79.5 ± 1.16	69.3 ± 2.33	0.3 ± 0.02	0.3 ± 0.01
M+AtFAD3	14.1 ± 0.93	17.9 ± 0.32	1.1 ± 0.07	5.2 ± 0.31			4.9 ± 0.33	4.5 ± 0.13	0.7 ± 0.06	2.9 ± 0.13	78.9 ± 1.40	69.2 ± 0.91	0.2 ± 0.01	0.3 ± 0.03
M+AtFAD3+GmCb5-A1	18.9 ± 1.05	21.5 ± 0.07	3.3 ± 0.33	9.8 ± 1.20			7.6 ± 0.56	6.0 ± 0.15	2.3 ± 0.26	6.1 ± 0.61	57.4 ± 2.78	42.5 ± 2.25	10.4 ± 0.70	14.0 ± 0.48
M+AtFAD3+GmCb5-C2	20.9 ± 0.38	21.7 ± 0.94	2.6 ± 0.05	10.1 ± 1.27			7.5 ± 0.17	5.1 ± 0.25	1.7 ± 0.08	5.6 ± 0.70	63.5 ± 0.63	53.8 ± 3.57	3.9 ± 0.05	3.8 ± 0.41
M+AtFAD3+GmCb5-C3	21.0 ± 0.21	23.2 ± 0.36	2.6 ± 0.07	12.8 ± 0.65			7.5 ± 0.14	5.5 ± 0.09	1.7 ± 0.02	7.2 ± 0.33	64.6 ± 0.45	49.4 ± 1.51	2.6 ± 0.28	2.0 ± 0.10
M+AtFAD3+GmCb5-E1	22.7 ± 0.78	23.2 ± 0.91	3.7 ± 0.49	11.6 ± 0.67			8.9 ± 0.32	5.8 ± 0.30	2.5 ± 0.29	7.2 ± 0.55	45.1 ± 4.33	38.5 ± 2.97	17.1 ± 2.78	13.8 ± 2.58

For Cb5 and FAD3 co-expression study 18:2 were added to the culture before induction with galactose. FAMES were analyzed by GC-FID. The induction time for culture maintained at 28^{0}C and 15^{0}C were 48 and and 96 hour respectively. Values represents mol percentage of total fatty acids. ± represents SD of three independent cultures. At= Arabidopsis thaliana; Gm= Glycine max; Cb5= Cytochrome b5; W= wild type yeast; M= mutant yeast disrupted in endogenous Cb5 gene; ND= Not detected.
