# Supplementary material for: Higher Plant Cytochrome b5 Polypeptides Modulate Fatty Acid Desaturation
Source: PLoS One. 2012 Feb 23;7(2):e31370. doi: 10.1371/journal.pone.0031370 (PMC3285619; doi:10.1371/journal.pone.0031370)
Supplement: Table S5 — Primer used for cloning of Cb5 , FAD2 and FAD3 of soybean (A) and Arabidopsis (B). (PDF) [file pone.0031370.s006.pdf]

Table S5. Primer used for cloning of Cb5, FAD2 and FAD3 of soybean (A) and *Arabidopsis* (B) .

Genes	Forward	Reverse
**(A)**		
Cb5-A1	AATTGGATCCATGGCTTCAGATCGG	AATTAAGCTTCTACTCTTTCTTGGTGTAGTG
Cb5-C2	AATTGGATCCATGGGTTCAAAAACCAAGAC	AATTAAGCTTTCAATTTTCTGACTCAGTG
Cb5-C3	AATTGGATCCATGGCCTCAAATCCCAAAAC	AATTAAGCTTTCATGAGGTGCTAGCATG
Cb5-E1	AATTGTCGACATGGGTGGGGAGCGGAAC	AATTAAGCTTTTATGTTGATTTGGTGTAGAAAC
FAD2-1B	AATTATCGATATGGGTCTAGCAAAGGAAAC	AATTGAGCTCTCAATACTTGTTCCTGTAC
FAD3-1A	AATTATCGATATGGTTAAAGACACAAAGC	AATTGAGCTCTCAGTCTCGGTGCGAGTG
**(B)**		
Cb5-A	AATTGGATCCATGTCTTCAGATCGGAAG	AATTAAGCTTCTAGTCTTTCTTGGTATAGTG
Cb5-B	AATTGGATCCATGGGCGGAGACGGA	AATTAAGCTTTCAAGAAGAAGGAGCCTTG
Cb5-C	AATTGGATCCATGGCGAATCTAATTTCG	AATTAAGCTTGTTATTACCAACAAACG
Cb5-E	AATTGGATCCACAAGAATCAAACAAACA	AATTAAGCTTACTTGAATCTTTCTCTC
FAD2	AATTGCGGCCGCATGGGTGCAGGTGGAAGAATG	AACCTTAATTAATCATAACTTATTGTTGTACCAG
FAD3	AATTGCGGCCGCATGGTTGTTGCTATGGACCAAC	AACCTTAATTAATTAATTGATTTTAGATTTGTC

The underlined nucleotides indicate restriction sites to facilitate cloning into yeast expression vector pESC (Stratagene).
